# 中国非吸烟人群被动吸烟与肺癌关系的*meta*分析

**DOI:** 10.3779/j.issn.1009-3419.2010.06.010

**Published:** 2010-06-20

**Authors:** 辉 赵, 俊东 谷, 洪瑞 许, 炳军 杨, 友奎 韩, 莉 李, 树忠 刘, 红 姚

**Affiliations:** 300121 天津，天津市人民医院胸外科 Department of Thoracic Surgery, Tianjin People's Hospital, Tianjin 300121, China

**Keywords:** 肺肿瘤, 被动吸烟, *meta*分析, Lung neoplasms, Passive smoking, *meta*-analysis

## Abstract

**背景与目的:**

本研究旨在探讨中国非吸烟人群被动吸烟与肺癌的关系。

**方法:**

通过计算机检索Medline、PubMed、CENTRAL（the Cochrane central register of controlled trials）、中国生物医学文献数据库系统（CBM）、中国期刊全文数据库（CNKI）、中文科技期刊全文数据库（VIP）等收集国内外1987年-2007年间公开发表的关于中国非吸烟人群被动吸烟与肺癌的研究文献，应用统计软件Stata 11.0进行数据分析，计算其合并优势比（odds ratio, OR）和95%置信区间（confidence interval, CI）。采用*Begg*和*Egger*法对发表偏倚进行量化检测。

**结果:**

纳入分析的文章共有16篇，合并分析结果表明：中国非吸烟人群被动吸烟与肺癌的关系有统计学意义（OR=1.13, 95%CI: 1.05-1.21, *P*=0.001）。每日被动吸烟≥20支、成年时期被动吸烟、非吸烟女性被动吸烟、被动吸烟暴露于工作环境等与肺癌的发生关系具有统计学意义，*P*值、OR值及95%CI分别为：*P*=0.000 3、OR=1.78、95%CI: 1.30-2.43，*P*=0.000 1、OR=1.50、95%CI: 1.23-1.83，*P*=0.000 7、OR=1.50、95%CI: 1.19-1.90，*P* < 0.000 1、OR=1.41、95%CI: 1.19-1.66。

**结论:**

中国非吸烟人群中，被动吸烟是肺癌发生的一个重要危险因素，尤其是暴露量≥20支/日、成年时期被动吸烟、女性、工作环境的被动吸烟与肺癌的发生关系密切。

## Abstract (en)

**Background and objective:**

Studies of passive smoking exposure in China however are of particular interest, because of the high lung cancer rate in people who are mostly non-smokers. The aim of this study is to explore the relationship between passive smoking and lung cancer among non-smoking Chinese.

**Methods:**

By searching Medline, PubMed, CENTRAL (the Cochrane central register of controlled trials), CBM, CNKI and VIP, *et al*, we collected both domestic and overseas published documents between 1987 and 2007 on passive smoking and lung cancer among non-smoking Chinese. Random or fixed effect models were applied to conduct *meta*-analysis on the case control study results, and the combined odds ratio (OR) and the 95% confidence interval (CI) were calculated as well.

**Results:**

Sixteen documents were included into the combined analysis, which indicated that there was statistical significance between passive smoking and lung cancer (OR=1.13, 95%CI: 1.05-1.21, *P*=0.001). It was significant of lung cancer among non-smoking subjects associated with amount of tobacco passively smoked more than 20 cigarettes daily, with life period in adulthood passive smoking exposure, with gender female, and with exposure to workplace. The P value, OR and 95%CI were *P*=0.000 3, OR=1.78, 95%CI: 1.30-2.43; *P*=0.000 1, OR=1.50, 95%CI: 1.23-1.83; *P*=0.000 7, OR=1.50, 95%CI: 1.19-1.90; *P* < 0.000 1, OR=1.41, 95%CI: 1.19-1.66; respectively. And there was no significant difference between passive smoking and lung cancer with amount of tobacco passively smoked within 20 cigarettes daily, with life period in childhood passive smoking exposure, with gender male and with exposure to spouse and parents.

**Conclusion:**

Passive smoking is an important risk factor of lung cancer among non-smoking Chinese, and for non-smoking women who expose to environment tobacco smoke in a long period of time have a close relationship with lung cancer risk.

当前在世界范围内，每年大约有超过一百万人死于肺癌，肺癌已成为肿瘤中导致男性死亡的第一杀手，在女性则为第二位^[1]^。肺癌已经成为一个重要的健康问题，它与吸烟有很重要关系，大约90%的肺癌均与烟草的暴露有关^[2]^。发达国家吸烟的人数比例正在下降，然而在发展中国家吸烟的比例却在上升，加之肺癌的发生率在非吸烟人群中也在增加，这就使肺癌成为一个重要的健康问题^[1]^。肺癌死亡率高、病因复杂，非吸烟人群被动吸烟与肺癌发生关系是目前研究的焦点之一。自20世纪80年代以来，国内外进行了大量关于被动吸烟与肺癌关系的研究，但研究结果并不完全一致。为此，本研究选择1987年-2007年公开发表的资料进行*meta*分析，从循证医学的角度探讨非吸烟人群被动吸烟与肺癌发生的关系。

## 1 材料与方法

### 1.1 资料来源

通过计算机检索Medline、PubMed、CENTRAL、中国生物医学文献数据库系统（CBM）、中国期刊全文数据库（CNKI）、中文科技期刊全文数据库（VIP）等，收集国内外1987年-2007年间公开发表的关于中国非吸烟人群被动吸烟与肺癌关系的研究文献。

### 1.2 文献纳入标准

#### 1.2.1 研究类型

病例对照研究。

#### 1.2.2 研究对象

病理证实或临床诊断为肺癌的中国非吸烟患者。

#### 1.2.3 研究方法

各文献研究假设及研究方法相似。

#### 1.2.4 原始数据

原文献提供的原始数据能够进行OR值及95%CI计算。

#### 1.2.5 划分标准

暴露及各因素分层划分标准基本相似。

### 1.3 文献排除标准

文献质量较差；未能提供原始数据用以计算OR值及95%CI值或非病例对照研究；研究人群为非中国肺癌患者等。

### 1.4 纳入文献的质量评价

根据“观察性流行病学研究报告规范（STROBE）——病例-对照研究”进行了量化评价（http://strobe-statement.org/index.php?id=availablechecklists）。

### 1.5 统计分析

阅读文献，按照*meta*分析要求整理数据，建立数据库，并核校数据。用优势比（odds ratio, OR）反映被动吸烟与肺癌发生关系的效应大小。所有数据分析采用Stata 11.0统计软件完成，每个分析采用*I*^2^统计量、*Galbraith*及*L’Abbe*作图法进行异质性检验，如果是同质的则采用固定效应模型（fixed effect model）分析，反之，则用DerSimonian Laird法随机效应模型（random effect model）分析，并对可能导致异质性的因素进行亚组分析或敏感性分析。采用*Begg*和*Egger*法对发表偏倚进行量化检测。以*P* < 0.05为差异具有统计学意义。

## 2 结果

### 2.1 文献基本情况

纳入本次*meta*分析的16篇文献的基本情况见表 1。其中病例组3 583例，对照组5 570例，纳入研究的每篇文献均提供了病例组和对照组的原始数据。

**1:** 纳入研究的基本特征 General characteristics of included trials

First author	Cases	Controls	Study type	OR	95%CI	Adjusted OR	Adjusted 95%CI	Years of data Collection	Year of publicaion	Region
HU^[3]^	104	163	Case-control	2.86	1.72-4.76	—	—	1990-1993	1996	Guangdong
ZHENG^[4]^	94	259	Case-control	1.04	0.61-1.78	1.04	0.59-1.85	1990-1993	1997	Peking
Zhong^[5]^	504	601	Case-control	1.15	0.85-1.57	1.1	0.8-1.5	1992-1994	1999	Shanghai
Gao^[6]^	246	375	Case-control	1.19	0.82-1.73	1.30	0.87-1.94	1984-1986	1987	Shanghai
Ko^[7]^	105	105	Case-control	0.86	0.46-1.60	0.8	0.4-1.6	1992-1993	1997	Taiwan
Wu-Williams^[8]^	417	602	Case-control	0.78	0.56-1.10	0.70	0.60-0.90	1985-1987	1990	Shenyang
Wang^[9]^	228	521	Case-control	1.39	0.88-2.20	1.19	0.7-2.0	1994-1998	2000	Gansu
McGhee^[10]^	324	763	Case-control	1.62	1.24-2.13	1.39	1.03-1.88	1983-1986	2005	Hong Kong
Liu^[11]^	54	202	Case-control	0.74	0.32-1.69	0.77	0.30-1.96	1985-1986	1991	Xuanwei
Lam^[12]^	199	335	Case-control	1.65	1.16-2.35	—	—	1983-1986	1987	Hong Kong
Lei^[13]^]	75	128	Case-control	1.19	0.66-2.16	—	—	—	1996	Guangzhou
SUN^[14]^	230	230	Case-control	4.18	2.14-8.16	2.86	1.69-4.84	1985-1991	1995	Haerbin
Lee^[15]^	268	445	Case-control	1.88	1.36-2.60	—	—	1992-1998	2000	Taiwan
LIU^[16]^	498	595	Case-control	1.93	1.31-2.83	1.65	1.10-2.47	1992-1993	2001	Shanghai
SONG^[17]^	115	124	Case-control	2.31	1.36-3.90	—	—	—	1999	Haerbin
LIN^[18]^	122	122	Case-control	1.20	0.60-2.39	—	—	1985-1990	1994	Haerbin
Combined	3 583	5 570		1.13	1.05-1.21					

### 2.2 纳入研究的质量评价

根据“观察性流行病学研究报告规范（strengthening the reporting of observational studies in epidemiolog, STROBE）——病例-对照研究”进行了量化评价，总体上英文文献质量好于中文文献（表 2）。

**2:** 纳入研究16篇文献的质量评价（STROBE声明） STROBE Statement-checklist criteria included in 16 reports of passive smoking and lung cancer risk

Items	Recommendations	Number of study[*n* (%)]
Title and abstract	1.1 Indicate the study’s design with a commonly used term in the title or the abstract	4 (25%)
	1.2 Provide in the abstract an informative and balanced summary of what was done	12 (75%)
Introduction		
Background/rationale	2 Explain the scientific background and rationale for the investigation being reported	13 (81.3%)
Objectives	3 State specific objectives, including any prespecified hypotheses	14 (87.5%)
Methods		
Stydy desin	4 Present key elemens of study design early in the paper	12 (75%)
Setting	5 Describe the setting, locations, and relevant dates, including periods of recruitment,	16 (100.0%)
Participants	6.1 Give the eligibility criteria, and the sources and methods of ascertainment, and	11 (68.6%)
	6.2 For matched studies, give matching criteria and the number of controls per case	9 (56.3%)
Variables	7 Clearly define all outcomes, exposures, predictors, potential confounders, and effect	8 (50.0%)
Datasources/	8 For each variable of interest, give sources of data and details of methods of	10 (62.5%)
Bias	9 Describe any efforts to address potential sources of bias	2 (12.5%)
Study size	10 Explain how the study size was arrived at	0 (0)
Quantitative variables	11 Explain how quantitative variables were handled in the analyses. If applicable,	9 (56.3%)
	12.1 Describe all statistical methods, including those used to control for confounding	14 (87.5%)
	12.2 Describe any methods used to examine subgroups and interactions	11 (68.8%)
Statistical methods	12.3 Explain how missing data were addressed	4 (25%)
	12.4 If applicable, explain how matching of cases and controls was addressed	6 (37.5%)
	12.5 Describe any sensitivity analyses	4 (25%)
Results		
	13.1 Report numbers of individuals at each stage of study-eg numbers potentially	13 (81.3%)
Participants	13.2 Give reasons for non-participation at each stage	8 (50%)
	13.3 Consider use of a flow diagram	0 (0)
Descriptive data	14.1 Give characteristics of study participants (eg, demographic, clinical, social) and	11 (68.8%)
	14.2 Indicate number of participants with missing data for each variable of interest	5 (31.3%)
Outcome data	15 Report numbers in each exposure category, or summary measures of exposure	13 (81.3%)
	16.1 Give unadjusted estimates and, if applicable, confounder-adjusted estimates	12 (75%)
	16.2 Report category boundaries when continuous variables were categorized	11 (68.8%)
	16.3 If relevant, consider translating estimates of relative risk into absolute risk for a	3 (18.8%)
Other analyses	17 Report other analyses done-eg analyses of subgroups and interactions, and	5 (31.3%)
Discusion		
Key results	18 Summarise key results with reference to study objectives	16 (100%)
Limitations	19 Discuss limitations of the study, taking into account sources of potential bias or	9 (56.3%)
Interpretation	20 Give a cautious overall interpretation of results considering objectives, limitations,	11 (68.8%)
Generalisability	21 Discuss the generalisability (external validity) of the study results	5 (31.3%)
Other information		
Funding	22 Give the source of funding and the role of the funders for the present study and,	2 (12.5%)
	if applicable, for the original study on which the present article is based	

### 2.3 各独立研究结果的异质性检验

针对被动吸烟这一因素对纳入研究的16篇文献分别采用*I*^2^统计量、*Galbraith*及*L’Abbe*作图法进行异质性检验，检验结果*I*^2^=27.3% < 50%；*Galbraith*图中各个研究均位于95%CI内（图 1）；*L’Abbe*图中各研究呈线性分布且均在该线周围（图 2）。可以认为各独立研究结果之间不存在明显的异质性，合并分析采用固定效应模型。

**1:**
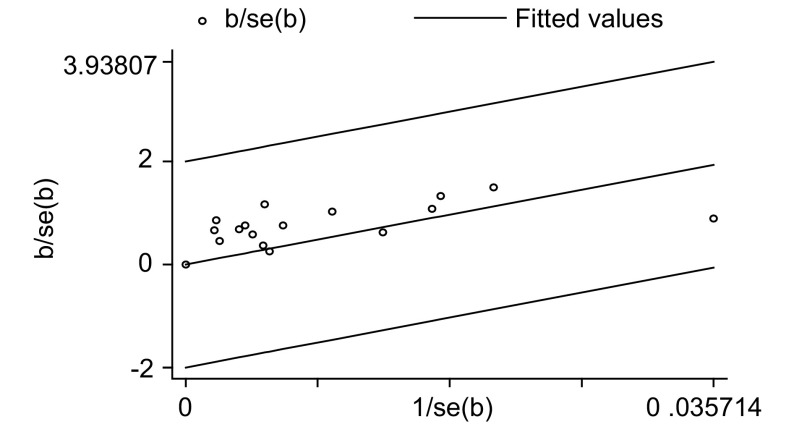
被动吸烟与肺癌关系的*Galbraith*图 *Galbraith* plot for lung cancer among non-smokers associated with passive smoking

**2:**
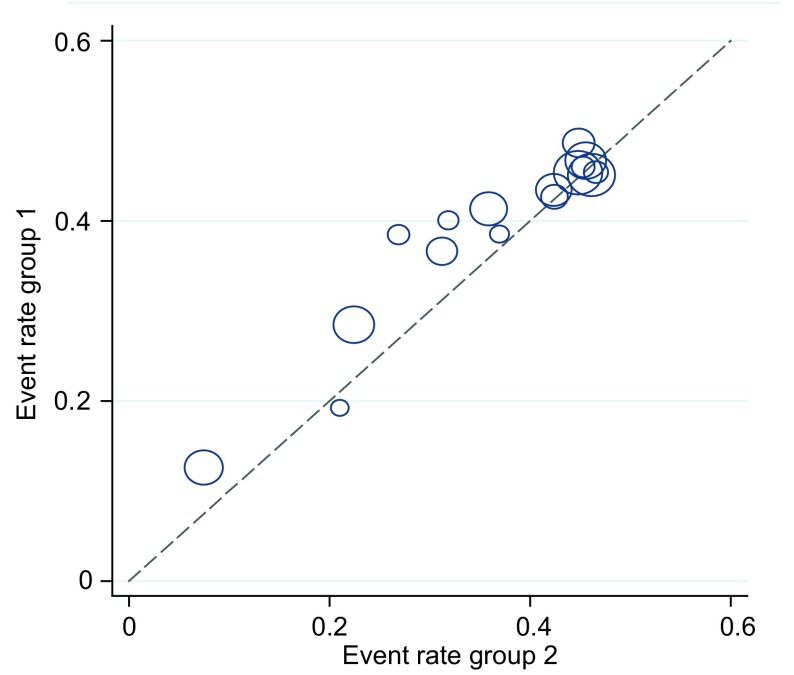
被动吸烟与肺癌关系的*L’Abbe*图 The *L'Abbe* plot for lung cancer among non-smokers associated with passive smoking

### 2.4 计算合并后综合效应的大小

结果显示非吸烟人群被动吸烟与肺癌存在一定的关系，合并OR值为1.13（95%CI: 1.05-1.21）。总体效应检验结果，*Z*=3.32，*P*=0.001（图 3）。

**3:**
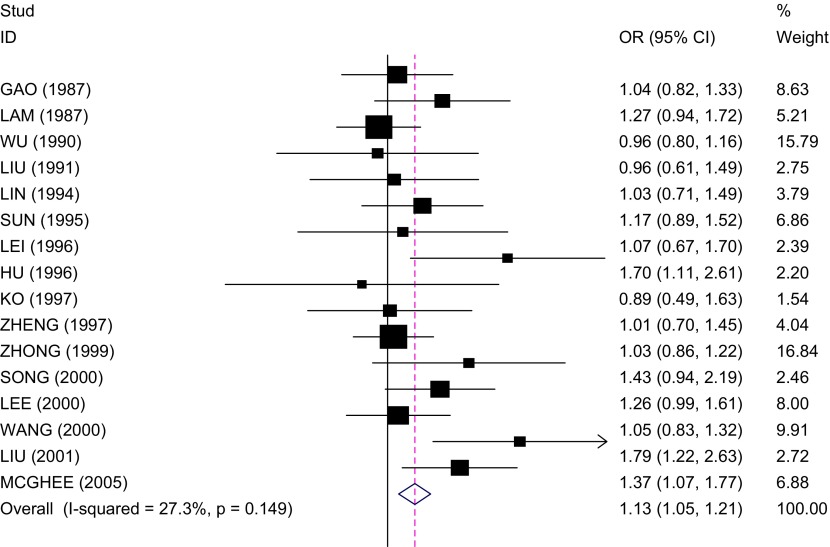
中国非吸烟人群被动吸烟与肺癌关系的森林图 Forest plot of odds ratios (OR) and 95% confidence intervals (CI) of lung cancer among non-smokers exposed to passive smoking

### 2.5 发表性偏倚的识别及敏感性分析

*Begg*和*Egger*法量化检测发表偏倚以及绘制的漏斗图显示，*Begg*’ test中*Pr*>|*z*|=0.300>0.05，图中各点沿中间水平线均匀分布，基本位于预计95%CI内；*Egger*’ test中*t*=1.42，*P*=0.178>0.05，95%CI为-0.63-3.11，包括0在内（图 4）。对纳入研究的16篇文献进行敏感性分析，剔除任意一篇文献后*meta*分析的OR均位于1.06-1.08之间，剔除前后未发生明显变化（图 5）。

**4:**
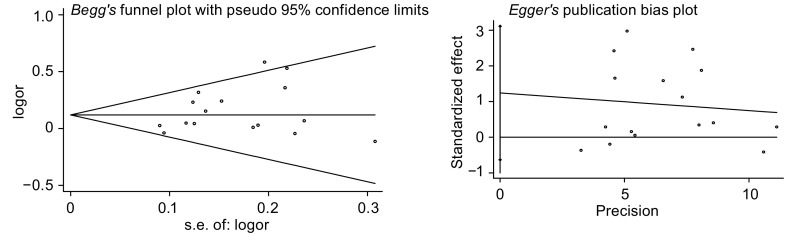
*Begg*和*Egger*法检测被动吸烟与肺癌关系的漏斗图 The funnel plot for lung cancer among non-smokers associated with passive smoking through *begg*'s and *Egger*'s test

**5:**
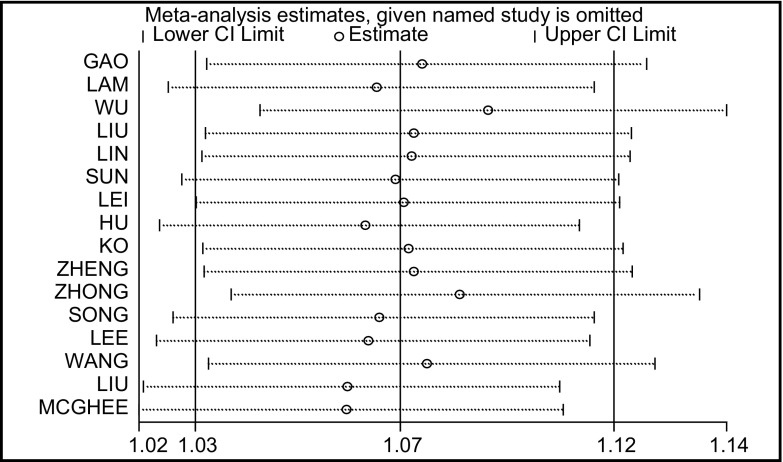
各篇文献对*meta*分析结果的影响 The influence of each document for the outcome of the *meta* - analysis

### 2.6 分层分析（表 3）

**3:** 不同类别分层非吸烟人群被动吸烟与肺癌的关系 Odds ratios (OR) and 95% confidence intervals (CI) of lung cancer among non-smokers associated with different categories

Categories	References No.	Combined OR	95%CI	*Z*	*P*
Amount of daily					
passive smoking					
< 20 cigarettes/day	3, 5, 12, 13	1.49	0.88-2.51	3.11	0.14
≥20 cigarettes/day	3, 5, 12, 13	1.78	1.30-2.43	3.61	0.000 3
Life period passive					
smoking exposure					
Exposure in childhood	5, 9, 15	1.42	0.97-2.08	3.87	0.08
Exposure in adulthood	5, 9, 15	1.50	1.23-1.83	4.00	0.000 1
Gender					
Male	4, 9, 10	1.36	0.93-2.00	1.58	0.11
Female	4, 5, 6, 7, 8, 9, 10 12, 14, 15, 16, 17	1.50	1.19-1.90	6.14	0.000 7
Sources of passive smoke					
exposure					
Spouse	6, 7, 8, 15	1.18	0.80-1.74	1.17	0.41
Parents	7, 8, 15	1.04	0.86-1.27	0.43	0.67
Workplace	7, 8, 14, 15, 16	1.41	1.19-1.66	4.00	< 0.000 1

#### 2.6.1 每日被动吸烟量与肺癌关系的综合效应大小

将每日被动吸烟量按 < 20支/日、≥20支/日进行分层，纳入分析的文献有4篇^[3, 5, 12, 13]^，经合并后OR值分别为1.49（95%CI: 0.88-2.51, *P*=0.14）和1.78（95%CI: 1.30-2.43, *P*=0.000 3）。

#### 2.6.2 不同生长时期被动吸烟与肺癌关系的综合效应大小

将被动吸烟人群按儿童时期、成年时期分层，纳入分析的文献有3篇^[5, 9, 15]^，经*meta*分析后得到其合并OR值分别为1.42（95%CI: 0.97-2.08, *P*=0.08）和1.50（95%CI: 1.23-1.83, *P*=0.000 1）。

#### 2.6.3 不同性别被动吸烟与肺癌关系的综合效应大小

将被动吸烟人群按男性、女性分层，纳入分析的文献有12篇^[4-10, 12, 14-17]^，经*meta*分析后得到其合并OR值分别为1.36（95%CI: 0.93-2.00, *P*=0.11）和1.50（95%CI: 1.19-1.90, *P*=0.000 7）。

#### 2.6.4 不同暴露来源非吸烟人群被动吸烟与肺癌关系的综合效应大小

将被动吸烟人群按暴露于丈夫、父母、工作环境分层，纳入分析的文献有6篇^[6-8, 14-16]^，经*meta*分析后得到其合并OR值分别为1.18（95%CI: 0.80-1.74, *P*=0.41）、1.04（95%CI: 0.86-1.27, *P*=0.67）和1.41（95%CI: 1.19-1.66, *P* < 0.000 1）。

## 3 讨论

环境烟草烟雾（environment tobacco smoke, ETS）是从燃烧的烟草释放出的侧烟流和吸烟者呼出的烟雾所构成，二者分别占80%和20%。吸烟者吸入的烟雾称之为主流烟雾。环境烟草烟雾的其它成分包括在喷烟时从燃烧的烟头逸出的烟雾和通过卷烟纸所弥散出的气体成分。这些成分被周围空气稀释，一旦被吸入，尤其是被非吸烟者吸入时，即称为被动吸烟。被动吸烟可能导致肺癌的基本原理与吸烟相似，ETS中含有各种有毒物质，包括诱变剂和致癌源如亚硝酸、4-氨基联苯、苯丙芘等。这些有害化学物质在ETS中比在烟草燃烧释放的主流烟雾中还要高。

非吸烟者肺癌在流行病学、危险因素及预后方面有其独特的生物学特征，已经成为肺癌独立的亚型，受到越来越多的关注^[19]^。既往的一些*meta*分析^[20-22]^结果表明，非吸烟人群被动吸烟与肺癌的发生有一定的关系。本次*meta*分析也证实非吸烟人群的被动吸烟是肺癌发生的危险因素（合并OR值=1.13，95%CI：1.05-1.21）。分别根据每日被动吸烟量、不同生长时期、不同性别以及不同暴露来源非吸烟人群被动吸烟进行分层分析后发现，每日被动吸烟量≥20支/日、成年期被动吸烟、非吸烟女性、工作环境的被动吸烟与肺癌的发生关系密切。

对纳入*meta*分析的16篇文献进行异质性检验发现，纳入研究的文献无论是*I*^2^统计量检验或*Galbraith*及*L’Abbe*作图法检验均不存在明显的异质性，说明文献的一致性较好，*meta*分析的结果更为可靠。敏感性分析也显示，剔除纳入研究的任一篇文献后合并效应量（OR）仍未发生明显变化（范围为1.06-1.08），提示该*meta*分析所得结论的稳定性较好。*Begg*和*Egger*两种检验方法对发表偏倚进行检测的结果也提示不存在明显的发表偏倚。本次*meta*分析纳入研究的人群均为中国的非吸烟者，这在一定程度上减小了由于人种、生活方式及饮食习惯不同造成的选择性偏倚，使得*meta*分析的研究结果更为可靠。但由于病例对照研究的特点，研究对象对烟草的被动暴露情况的回忆可能存在一定的不准确，故难以避免由此造成的回忆偏倚。

尽管该*meta*分析结果显示被动吸烟只是轻微增加非吸烟人群患肺癌的风险（OR=1.13），但由于中国吸烟人群数量巨大，在很多公共场所造成烟草环境污染，致使许多非吸烟者成为被动吸烟的受害者，因此在中国被动吸烟已成为肺癌发生的一个重要的危险因素，同时也提示有关部门有必要进一步采取措施对公共场所的烟草环境进行干预。
